# Analysis of Candidozyma auris – an emerging threat in intensive care units

**DOI:** 10.3205/dgkh000598

**Published:** 2025-11-28

**Authors:** Akshata Uppar, Saravana Priya Jayakumar Kalpana, Veena Kumari Haradara Bahubali

**Affiliations:** 1 National Institute of Mental Health and Neurosciences (NIMHANS) Bengaluru, Karnataka, India

**Keywords:** Candidozyma auris, Candida auris, antifungal susceptibility, central venous catheter, colonization, critically ill, emergent pathogen, ICU, infection control, multidrug resistance, nosocomial outbreak

## Abstract

**Introduction:**

*Candidozyma (C.) auris* (formerly *Candida auris*) is an emerging fungus posing a serious global health threat due to its multidrug resistance and difficulty in identification with standard laboratory methods. It causes severe illness in hospitalized patients, with risk factors including recent surgery, diabetes, and the use of broad-spectrum antibiotics and antifungals. Colonization of the respiratory tract, catheters, and skin with *C. auris* can lead to serious infections, particularly through central venous catheters, urinary catheters, and tracheostomy tubes, resulting in fungemia.

**Case series:**

: We present a case series of five ICU patients with fungemia caused by *C. auris*. The cases emphasize the immune status, screening, and clinical manifestations of these patients. Antifungal susceptibility testing using broth microdilution was performed, and antifungals were administered based on sensitivity reports. Three patients showed improvement with appropriate therapy.

**Conclusion:**

*C. auris* infections are increasing in prevalence and represent a significant burden to healthcare system and patients. Aggressive treatment and stringent infection control measures are essential to prevent the spread, multi-drug resistance, and mortality associated with *C. auris*. This study highlights the importance of effective treatment management, including the selection of antifungal agents and the implementation of robust infection control practices, to combat this emerging pathogen.

## Zusammenfassung

**Einleitung:**

*Candidozyma (C.) auris* (ehem. *Candida auris*) ist ein gefährlicher Hefepilz, der aufgrund seiner Multiresistenz und der Schwierigkeit, ihn mit Standard-Labormethoden zu identifizieren, eine ernsthafte globale Gesundheitsbedrohung darstellt. Er verursacht schwere Erkrankungen bei Krankenhauspatienten, zu deren Risikofaktoren kürzlich durchgeführte Operationen, Diabetes und die Verwendung von Breitbandantibiotika und Antimykotika gehören. Die Besiedlung der Atemwege, von Kathetern und der Haut mit *C. auris* kann zu schwerwiegenden Infektionen, insbesondere einer Fungämie, führen.

**Fallserie:**

Es wird eine Fallserie von fünf Intensivpatienten mit einer durch *C. auris* verursachten Fungämie vorgestellt. Die Fälle verdeutlichen den Immunstatus, das Screening und die klinischen Manifestationen. Die Empfindlichkeit gegenüber Pilzen wurde mit Hilfe der Mikrodilutionsmethode bestimmt, und die Antimykotika wurden auf der Grundlage der Empfindlichkeit verabreicht. Bei drei Patienten trat unter der entsprechenden Therapie eine Besserung ein.

**Schlussfolgerung:**

*C. auris*-Infektionen treten immer häufiger auf und stellen eine erhebliche Belastung für das Gesundheitssystem und die Patienten dar. Eine aggressive Behandlung und strenge Maßnahmen zur Infektionskontrolle sind unerlässlich, um die Ausbreitung, die Multiresistenz und die mit *C. auris* verbundene Sterblichkeit zu verhindern. Die Studie unterstreicht die Bedeutung eines effektiven Behandlungsmanagements einschließlich der Auswahl von Antimykotika und der Umsetzung robuster Infektionskontrollpraktiken, um diesen gefährlichen Erreger zu bekämpfen.

## Introduction

*Candidozyma (C.) auris* (formerly *Candida auris*), first reported in 2009 in Japan [1], has emerged as a significant nosocomial pathogen. It is associated with outbreaks in intensive care units (ICUs) and poses a major concern despite enhanced infection prevention and control (IPC) measures [2]. Variable antifungal susceptibility and resistance mechanisms further complicate its management [3]. The difficulty in identification using conventional and molecular techniques, coupled with its environmental niches and mechanisms of spread, has hindered the effective control of *C. auris* infections [4], [5].

The use of prophylactic antifungal agents such as fluconazole has led to an increased prevalence of colonization and infection with non-albicans *Candida* spp. in recent years [6]. Historically, invasive candidiasis was predominantly caused by *Candida alb*icans [7]. However, with the shift towards non-albicans *Candida* spp., fluconazole is no longer the mainstay of empirical antifungal treatment [8]. *C. auris*, with its ability to rapidly spread among critically ill patients, has become a dominant opportunistic pathogen in these individuals [9].

The primary objectives of this study are to investigate the prevalence and characteristics of *C. auris* infections in ICU settings, evaluate the effectiveness of antifungal treatment, and assess the impact of IPC measures on controlling its spread. By addressing these objectives, the study aims to provide insights into better management practices for this emerging pathogen.

## Case description

### Case 1

A 36-year-old male was admitted to the emergency department with subdural hematoma and temporal contusion. Following surgery, he was transferred to the ICU due to poor sensorium. A urinary catheter was in place since admission. Upon admission to the ICU, the patient continued to receive mechanical ventilation, and a central line was inserted on the 4^th^ day in the right internal jugular vein. Routine blood investigations, including hemogram, liver, and kidney function tests, were performed. Due to the failure to extubate and worsening chest condition, a CT scan revealed hemopneumothorax, and an implantable cardioverter-defibrillator was placed. The patient improved and was off the ventilator but developed fever. A paired blood culture identified *C. auris* by MALDI-TOF MS. The central line was removed on the 29^th^ day. The patient was diagnosed with *C. auris* fungemia and administered caspofungin (loading dose of 70 mg, followed by 50 mg/day). 72 hours after treatment, the patient improved, and a repeated blood culture yielded no growth. The patient was transferred out of the ICU, and discharged with a recommendation to continue caspofungin for 14 days from the last negative blood report. Total ICU days were 44, with 29 days of central line catheter use and 32 days of Foley’s catheter.

### Case 2

A 31-year-old male diagnosed with right fronto-temporo-parietal epidural hematoma (EDH) underwent surgery and was shifted to the ICU postoperatively due to failing saturation after EDH surgery. A urinary catheter was in place since admission, and a central line was inserted on the 3^rd^ day in the left internal jugular vein. The patient developed new onset weakness and underwent re-exploration surgery. Suspected pneumonia led to treatment with cefoperazone-sulbactam and amikacin due to endotracheal aspiration yielding Klebsiella spp. The patient was gradually weaned off the ventilator but continued to have fever spikes. Paired blood culture identified *C. auris*. The central line, in place for 10 days, was suspected of causing a catheter-related bloodstream infection (CRBSI). The patient was treated with caspofungin for 14 days with a loading dose of 70 mg, followed by 50 mg/day. The fever decreased, and repeated blood cultures showed no growth after 48 hours of therapy. The patient was discharged with advice to continue caspofungin for two weeks. Total ICU days were 17, with 10 days of central line catheter use, and 13 days of Foley’s catheter.

### Case 3

A 31-year-old male diagnosed with right fronto-temporo-parietal EDH underwent surgery. Postoperatively, the patient experienced dropped saturation and developed bilateral aspiration pneumonitis, leading to ICU admission. A central line was placed on the 3^rd^ day in the left internal jugular vein. Following re-exploration surgery, the patient was weaned off the ventilator and transferred to the normal ward. The patient developed fever, and a workup revealed *C. auris* in paired blood culture. The central line, in place for 10 days, was suspected of causing CRBSI. Caspofungin was administered for 14 days with loading dose of 70 mg, followed by 50 mg/day), reducing the fever. The patient was discharged, with follow-up blood cultures showing no growth. The patient had no significant medical history and was advised to continue caspofungin for 14 days from the date of the last blood culture showing no growth. Hematological, biochemical, and coagulation profiles were within normal limits. Total device days for the central line were 10, and total ICU days were 44 with 13 days on Foley’s catheter.

### Case 4

A 69-year-old male with a left temporal contusion and subarachnoid hemorrhage was admitted to the emergency department and underwent surgery. The patient remained on ventilation, with a urinary catheter and central line placed in the right internal jugular vein on the 3^rd^ day of ICU admission. The patient had continuous fever spikes and elevated total counts. Urine culture identified *C. auris* with no other positive sites during that period. The patient was not prescribed an antifungal. Blood culture confirmed fungemia after 7 days of incubation. The patient died on day 2 of ICU and day 10 of hospital stay. Total device days for the central line were 10, and total ICU days were 18 with 13 days on a Foley’s catheter.

### Case 5

A 67-year-old male diagnosed with multiple central contusions underwent surgery and was admitted to the ICU due to low Glasgow coma scale score. The patient remained on ventilation. Urine culture and subsequent urine culture sensitivity reports identified *C. auris* with no other positive sites during that period. The patient was not prescribed an antifungal. Blood culture confirmed fungemia after 7 days of incubation. The patient died before the blood culture report showed growth. Total device days for the central line were 12, and total ICU days were 20 with 15 days on a Foley’s catheter.

### Cross-sectional data

#### Identification of C. auris

Identification was performed using MALDI-TOF MS and ensuring accurate species identification.

#### Antifungal susceptibility testing

Susceptibility to fluconazole, itraconazole, posaconazole, voriconazole, amphotericin B, caspofungin, and anidulafungin was tested using the broth microdilution method.

#### Patient monitoring

Routine blood investigations, including hemogram, liver and kidney function tests, were conducted. To screen for *C. auris*, paired blood cultures, urine cultures, and ear swabs are collected using aseptic techniques and promptly transported to the lab. Proper labeling and documentation ensure accurate detection and timely treatment. 

#### Treatment protocol

Caspofungin was started empirically and continued based on sensitivity reports. Patients received a loading dose of 70 mg, followed by 50 mg/day, and adjusted as necessary based on clinical response.

#### Infection prevention and control (IPC) measures

These included isolation of infected patients, strict hand hygiene, and terminal cleaning of patient rooms with hypochlorite. Environmental screening was conducted to identify potential sources of transmission (Table 1 (Tab. 1)).

These methods ensure accurate identification, effective treatment, and prevention of *C. auris* transmission in ICU settings.

#### Antifungal susceptibility testing

All isolates were tested for susceptibility to fluconazole, itraconazole, posaconazole, voriconazole, amphotericin B, caspofungin, and anidulafungin (Table 2 (Tab. 2)). *C. auris* was predominantly found in immunocompromised patients, often as a skin or urinary tract colonization. Invasive infections occurred in critically ill patients in ICUs, with major risk factors including recent surgery, diabetes, and broad-spectrum antibiotic use. The mortality rate associated with these infections was above 50% [10]. 

### Summary of the case series

Out of 5 patients with *C. auris* infections (fungemia and CRBSI), 3 were treated with caspofungin and recovered, 2 patients who did not receive antifungal treatment expired (Table 3 (Tab. 3)).

## Discussion

This study presents a cluster of cases with probable acquisition of infection by case 1 from the environment of the critical care unit, and subsequent colonization and infection of the other patients in the following months. Out of 5 patients, 3 were treated with antifungals and recovered, 2 patients who did not receive antifungal treatment expired. 

*C. auris* is found mainly in immunocompromised patients in ICUs as a skin or urinary tract colonization. Invasive infection occurs in critically ill patients in intensive care units with major risk factors and are associated with a mortality rate above 50% [11], [12], [13], [14].

*C. auris* is capable of surviving on surfaces for extended periods. After seven days, approximately 38% of the yeast remains cultivable on dry surfaces, while about 93% persists on moist surfaces. On stainless steel with an initial inoculum of ~4.8 lg colony-forming units (cfu), approximately ~3.5 lg cfu remain after 4 days and ~0.4 lg cfu after 14 days. On plastic surfaces, biofilm formation with an initial inoculum of ~8 lg cfu results in ~4.3 lg cfu being recultivable after 14 days.

This highlights the importance of thorough and frequent cleaning in healthcare settings. Effective disinfection practices, using disinfectants proven effective against *C. auris*, are essential for preventing the spread of this resilient pathogen in such environments [15], [16].

Routine screening and surveillance are vital for early detection of *C. auris* colonization and infection. Protocols for screening healthcare workers and patients, especially in high-risk areas such as ICUs, should be implemented. Effective screening helps identify and isolate colonized patients, preventing further transmission [17]. Environmental sampling and screening of healthcare workers (HCWs) and all close contacts (patients in the same ICU) was carried out; the screening results were negative [18].

Patients colonized or infected with *C. auris* should be isolated in single rooms with dedicated bathroom facilities. Isolation helps contain the spread of the pathogen within healthcare settings. Managing isolated patients requires meticulous planning and adherence to infection control protocols [19].

The following measures were implemented: standard precautions including hand hygiene were strictly adhered to and all infected patients with *C. auris* were isolated in a single room, with the room terminally cleaned after discharge using a hypochlorite solution at 1,000 ppm of available chlorine (i.e., a concentration higher than that routinely used).

Hand hygiene is a cornerstone of infection control. HCWs adhered to strict hand hygiene protocols, including the use of alcohol-based hand rubs and regular hand washing. The importance of proper hand hygiene and the use of personal protective equipment cannot be overstated in preventing the transmission of *C. auris* [20].

All isolates were subjected to antifungal susceptibility testing against fluconazole, itraconazole, posaconazole, voriconazole, amphotericin B, caspofungin, and anidulafungin [21], [22].

Treating *C. auris* infections is challenging due to the increasing reports of echinocandin-resistant and pan-resistant strains. In this study, three out of five patients were treated with antifungals and recovered, while the two who did not receive antifungal treatment died. Early detection and appropriate antifungal therapy are essential for improving patient outcomes [23].

Managing *C. auris* clusters/outbreaks requires a coordinated approach, including contact tracing, patient transfers, and additional screening measures. Public health departments play a crucial role in monitoring outbreaks and implementing control measures. Reporting cases to state or local health authorities is necessary for effective outbreak management [24].

Patients often remain colonized with *C. auris* for long periods, even after treatment. Long-term infection control measures, including regular monitoring of colonized patients, are necessary to prevent recurrence and further spread [24].

Educating HCWs about *C. auris*, its transmission, and infection control measures is essential. Regular training sessions and updates on the latest guidelines and recommendations help ensure that HCWs are equipped to manage *C. auris* infections effectively [25], [26]. 

## Conclusion

*C. auris* infections are increasing in prevalence and represent a significant burden to the healthcare system and patients. An aggressive approach to treating *C. auris* in most patients is essential to prevent subsequent invasive spread, multi-drug resistance, and ultimately, mortality. To achieve this, it is imperative that detailed information regarding *C. auris* urinary and blood isolation, concomitantly positive sites, treatment management – including both infection control measures and antifungal agent selection – and duration of therapy, be collected and disseminated. This will enable the design of effective, evidence-based treatment regimens for patients infected with *C. auris*. Moving forward, a coordinated effort is required to address the challenges posed by *C. auris* and to implement effective strategies to manage and control its spread.

## Notes

### Authors’ ORCIDs

- Saravana Priya Jayakumar Kalpana: https://orcid.org/0000-0002-1143-8206

### Ethical approval 

The project was approved by the Institutional Ethics Committee and all patients gave written consent before participation after adequate explanation. Informed consent from patients was obtained, and careful measures were taken to maintain confidentiality regarding the identity of these patients.

### Funding

None. 

### Competing interests

The authors declare that they have no competing interests.

## Figures and Tables

**Table 1:**
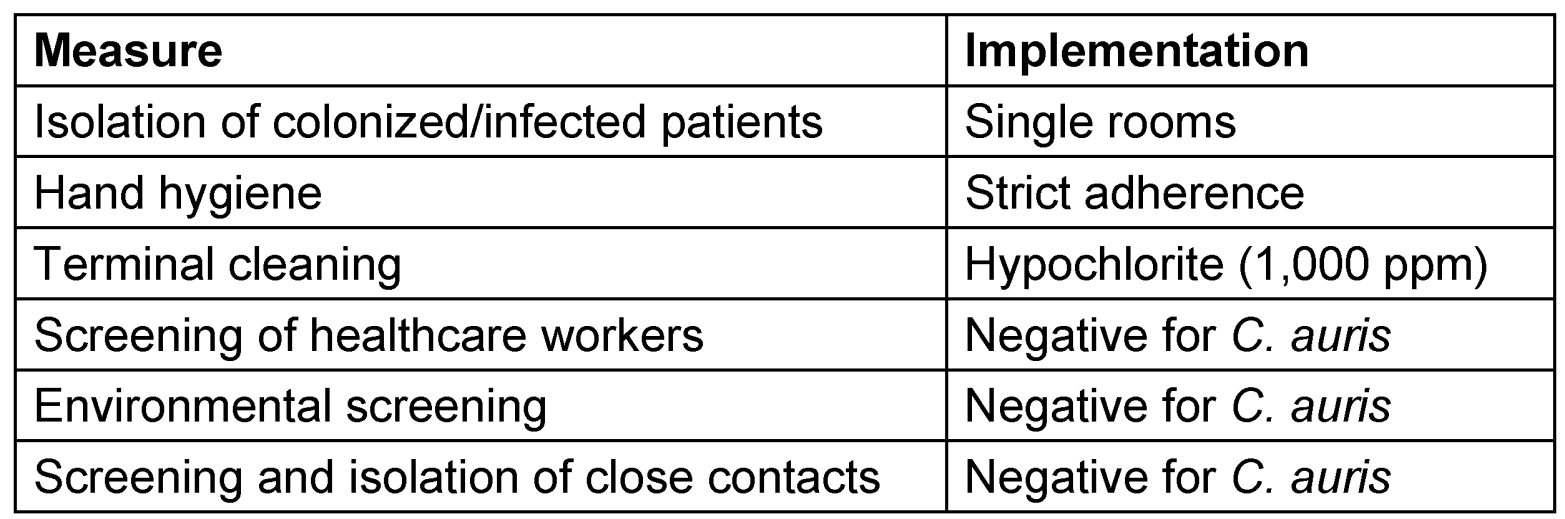
Infection control measures

**Table 2:**
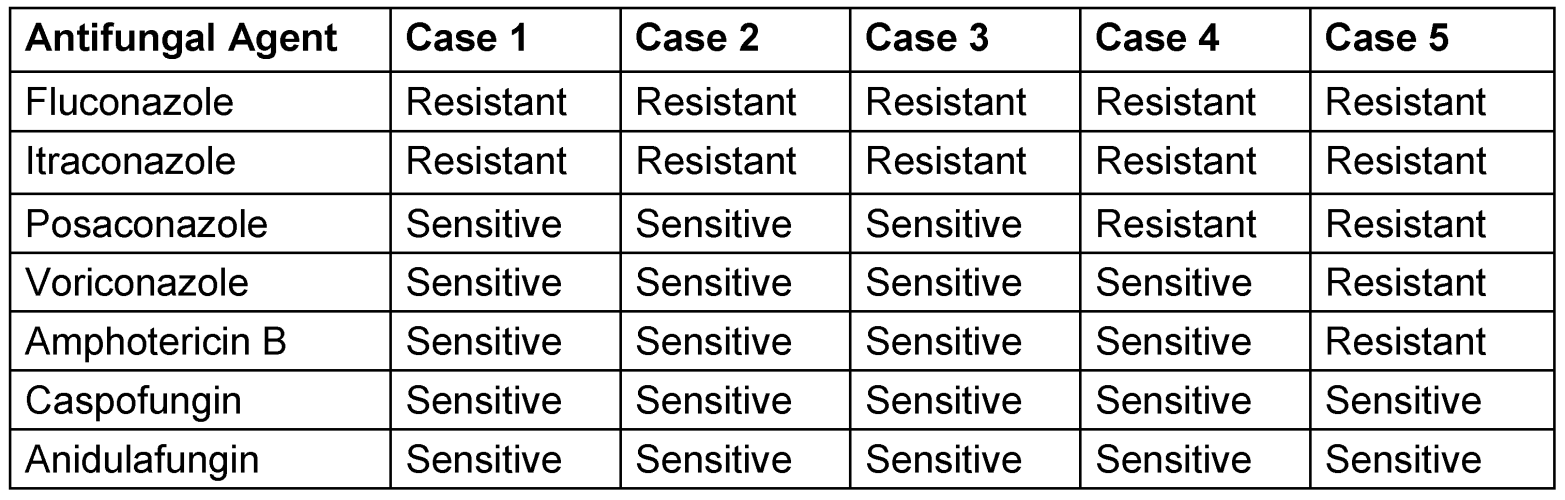
Antifungal susceptibility testing

**Table 3:**
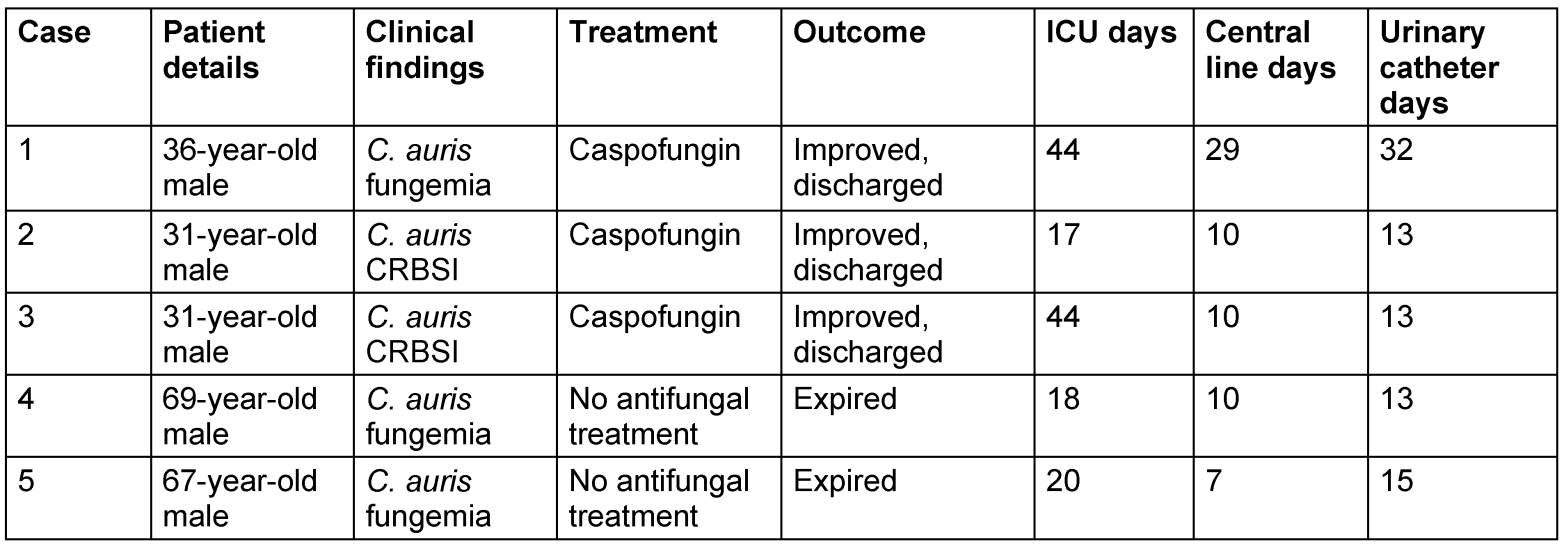
Comprehensive analysis of *C. auris* infections: clinical manifestations, therapeutic interventions, and hospitalization metrics
